# Metalaxyl Effects on Antioxidant Defenses in Leaves and Roots of *Solanum nigrum* L.

**DOI:** 10.3389/fpls.2017.01967

**Published:** 2017-11-30

**Authors:** Alexandra de Sousa, Hamada AbdElgawad, Han Asard, Ana Pinto, Cristiano Soares, Simão Branco-Neves, Teresa Braga, Manuel Azenha, Samy Selim, Soad Al Jaouni, Fernanda Fidalgo, Jorge Teixeira

**Affiliations:** ^1^BioSystems and Integrative Sciences Institute, Departamento de Biologia, Faculdade de Ciências, Universidade do Porto, Porto, Portugal; ^2^Laboratory for Molecular Plant Physiology and Biotechnology, Department of Biology, University of Antwerp, Antwerp, Belgium; ^3^Department of Botany and Microbiology, Faculty of Science, Beni-Suef University, Beni-Suef, Egypt; ^4^CIQ-UP, Departamento de Química e Bioquímica, Faculdade de Ciências, Universidade do Porto, Porto, Portugal; ^5^Department of Clinical Laboratory Sciences, College of Applied Medical Sciences, Aljouf University, Sakaka, Saudi Arabia; ^6^Department of Microbiology and Botany, Faculty of Science, Suez Canal University, Ismailia, Egypt; ^7^Department of Hematology and Yousef Abdullatif Jameel Chair of Prophetic Medicine Application (YAJCPMA), Faculty of Medicine, King Abdulaziz University, Jeddah, Saudi Arabia

**Keywords:** photosynthesis, sugar metabolism, antioxidant responses, ascorbate-glutathione cycle, fungicide

## Abstract

Overuse of pesticides has resulted in environmental problems, threating public health through accumulation in food chains. Phytoremediation is a powerful technique to clean up contaminated environments. However, it is necessary to unravel the metabolic mechanisms underlying phytoremediation in order to increase the efficiency of this process. Therefore, growth, physiological and biochemical responses in leaves and roots of *Solanum nigrum* L. exposed to the commonly used fungicide metalaxyl were investigated. This species shows characteristics that make it valuable as a potential tool for the remediation of organic pollutants. We found that once inside the plant, metalaxyl altered carbon metabolism, which resulted in a reduction of growth and lower biomass accumulation due to impairment of carbohydrate production (total soluble sugar, starch, rubisco) and increased photorespiration (glycolate oxidase, Gly/Ser ratio). A significant increase of antioxidant defenses (polyphenols, flavonoids, tocopherols, ascorbate, glutathione, superoxide dismutase, catalase, peroxidases, monodehydroascorbate- and dehydroascorbate reductase, gluthatione reductase) kept reactive oxygen species (ROS) levels under control (superoxide anion) leaving cell membranes undamaged. The results suggest that enhancing carbon assimilation and antioxidant capacity may be target parameters to improve this species’ phytoremediation capacities.

**Highlight**s

• Metalaxyl inhibits growth by reducing photosynthesis and inducing photorespiration

• Elevated antioxidant defenses protect metalaxyl-treated plants from oxidative damage

• Ascorbate and glutathione are key antioxidants in metalaxyl tolerance.

## Introduction

Pesticides are a commonly used to prevent crop losses to maintain high yields of economically important crops, to fulfill the rising demands in world’s food production (Gill and Garg, 2014). However, non-optimized use of pesticides carries risks and has led to a global pollution problem. Although pesticide commercialization is subject to strict legal restrictions accidental spills and leakages due to incorrect disposal and excessive use, pose a great threat to the environment and human health. These chemicals easily contaminate air, soils and groundwater, affecting non-target systems (Damalas and Eleftherohorinos, 2011). Moreover, dramatic increases in applied pesticide volumes decrease crop productivity and profitability on arable lands (Godfray et al., 2010). Damage of crops may occur even when recommend dosages are applied to crops, for example when pesticides drift away from target crops. Drift can account for a loss of 40–70% of non-targeted crops (Pimentel, 2009).

Although also fungicides are widely used in agriculture, their environmental effects have received less attention than herbicides and insecticides. Metalaxyl (C_15_H_21_NO_4_, methyl *N*-(methoxyacetyl)-*N*-(2,6-xylyl)-_D_-alaninate, CAS No. 57837-19-1) is the most active versatile and widely used phenylamine fungicide to control oomycetes pathogens on a wide range of crops, including potatoes, peas, tomatoes, tobacco, grape vines, maize and sorghum (Sukul and Spiteller, 2000). This fungicide is commercially available since 1979 and was chosen for this study, for its systemic and residual effects and its broad-spectrum activity, which are explained by its chemical properties (Yao et al., 2009). Its main chemical features can be summarized as: (a) high solubility in water which increases the risk to reach ground and surface water by agricultural run-offs and leakage through the soil profile, (b) moderate stability to hydrolysis under physiological pH, (c) moderate stability to photolysis, (d) low volatility and, (e) a high octanol water partition coefficient indicating a low tendency to be adsorbed by the soil matrix (Yao et al., 2009). Metalaxyl is also not very biodegradable and field dissipation occurs through aerobic microbial degradation, uptake by plants and leaching (Roberts and Hutson, 1998; Wilson et al., 2000; Sukul, 2006).

Metalaxyl is considered a threat to the environment as it is moderately carcinogenic and mutagenic to mammals and aquatic organisms dramatic. Increases in applied pesticide volumes, decrease crop productivity and profitability on arable lands (Hrelia et al., 1996; Abass et al., 2007). It contaminates groundwater and concentrations of up to 236 ppb have been detected (EPA, 1994). The EPA decided to initiate local educational programs if metalaxyl concentrations in groundwater reach 400 ppb. Therefore, the development of innovative and ecofriendly strategies for the remediation of this fungicide before it reaches food webs is imperative.

Phytoremediation has gained increasing attention and has become a rapidly expanding industry. It includes a vast group of phytotechnologies such as phytodegradation, phytovolatilization, phytostabilization, phytoextraction, and rhizofiltration, that use plants and their associated microbiota to remove heavy metals and organic pollutants from soils, sediments, water and air (Etim, 2012). Studies to explore the potential of various species for phytoremediation have uncovered pathways and genes involved in xenobiotic degradation. For example, a number of species (*Arabidopsis thaliana, Betula pendula, Fraxinus excelsior, Medicago sativa, Nicotiana tabacum, Panicum virgatum, Pinus nigra, Robinia pseudoacacia, Salix caprea, Solanum nigrum*), were found that successfully degrade a large variety of polychlorinated biphenyls (PCBs) in soil and water (Schell, 1985; Brazil et al., 1995; Siciliano and Germida, 1998; Narasimhan et al., 2003; Chekol et al., 2004; Villacieros et al., 2005; Leigh et al., 2006; Ionescu et al., 2009). Volatile organic compounds (VOCs) comprise a large family of molecules, including methyl tert-butyl ether (MTBE), trichloroethylene (TCE) and tetrachloroethene (PCE), major soil and groundwater, contaminants. Wang et al. (2010) showed that maize and wheat efficiently removed toluene from soil samples, while TCE was also efficiently removed by poplar and yellow lupin (Weyens et al., 2009, 2010a,b). Ryegrass and pea plants are principal species involved in hydrocarbon biodegradation (Andria et al., 2009; Germaine et al., 2009), while bentgrass is capable of trinitrotoluene remediation (Thijs et al., 2014). Nonetheless, to date only few reports have been published regarding metalaxyl phytoremediation, and only our research group addressed this issue with the well-known Cd and Zn hyper-accumulator weed, *S. nigrum* (black-nightshade) (Wilson et al., 2000, 2001). This annual or perennial plant has a ubiquitous distribution since it can adapt to several climates. *S. nigrum* plants display characteristics that make it an outstanding tool for phytoremediation, such as fast growth and large shoot biomass (Pilon-Smits, 2005). In fact, it has been reported that *S. nigrum* accumulates metalaxyl and hexachlorocyhexane in leaves, and that hairy root cultures of these plants can remediate up to 25 polychlorinated biphenyls congeners, revealing again its potential for phytoremediation of organic pollutants (Rezek et al., 2007; Teixeira et al., 2011). It is well known that response to biotic and abiotic stresses through the fast generation of reactive oxygen species (ROS), such as superoxide anion ($O_{2}^{•–}$), hydrogen peroxide (H_2_O_2_), singlet oxygen (^1^O_2_) and hydroxyl radicals (HO^.^), leading to oxidative stress (Das and Roychoudhury, 2016). ROS-induced imbalances often results in membrane oxidation and damage to DNA and proteins (Choudhury et al., 2017). To mitigate ROS effects, plants evolved an antioxidant defense machinery composed of enzymatic (e.g., SOD, CAT, APX) and non-enzymatic (e.g., ascorbate, glutathione, tocopherols, polyphenols) components (Choudhury et al., 2017). SOD (EC is a key antioxidant metalloenzyme widely distributed in all aerobic organisms. This enzyme catalyzes the removal of $O_{2}^{•–}$ by dismutating it into O_2_ and H_2_O_2._ Therefore, SOD reduces the risk of HO^.^ Formation *via* the Haber-Weiss reaction. CAT is an enzyme responsible for the direct dismutation of H_2_O_2_ into H_2_O and O_2_. It has a very huge turnover rate and does not require a reducing equivalent. CAT also has high affinity for H_2_O_2_, but lower specificity for organic peroxides. APX is a crucial enzyme involved in H_2_O_2_ detoxification from plant chloroplasts through the ascorbate-glutathione cycle. The APX reduces H_2_O_2_ to H_2_O and dehydroascorbate (DHA), using ASC as a reducing agent (Mittler et al., 2004; Gill and Tuteja, 2010). *In vitro* studies demonstrated that metalaxyl can be accumulated and/or degraded by *S. nigrum* cell suspension cultures only 5 h after exposure to the fungicide (de Sousa et al., 2013). Both *in vivo* and *in vitro* studies suggested that a suitable response of the antioxidant system is a major factor in metalaxyl tolerance in *S. nigrum* (Teixeira et al., 2011; de Sousa et al., 2013). Superoxide dismutase (SOD), catalase (CAT) and ascorbate peroxidase (APX) exhibited higher activity in cell suspension cultures of *S. nigrum* (de Sousa et al., 2013), while glutathione peroxidase (GPX) and glutathione-S transferase (GST) were enhanced (Teixeira et al., 2011). The recommended dose of metalaxyl in crop production is 600 g a.i ha^-1^ (0.8 mg kg^-1^ soil). Since the antioxidant response seems to be a key player in metalaxyl tolerance, this study is an expansion of our knowledge regarding oxidative metabolism with new information regarding carbon assimilation. Manipulation of these metabolic pathways may be key to improve *S. nigrum*’s remediation capacities. Attention was directed to enzymes and metabolites involved in the ascorbate-glutathione cycle and non-antioxidant metabolites (phenols, flavonoids and tocopherols), to further dissect the response of *S. nigrum* to metalaxyl. Since metalaxyl may have a negative impact on carbon metabolism leading to a reduction in growth and biomass, sugar accumulation (total soluble sugar, starch) and photosynthetic/photorespiration-related enzymes [rubisco and glycolate oxidase (GO)] were also analyzed.

## Materials and Methods

### Experimental Design

Seeds of *S. nigrum* were collected from plants grown in Campo Alegre, Porto, Portugal (41°9′55, 79″N and 8°38′19, 48″O) and surface sterilized with 70% (v/v) ethanol for 3 min, followed by 20% (v/v) sodium hypochlorite containing 0.02% (v/v) of Tween-20 for 3 min. Then, the seeds were washed in sterile deionized water in 3 cycles of 1 min each, and placed in filter papers in a flow hood until the seeds and the filter papers were completely dried. Seeds were placed in sterile Petri dishes (diameter 10 cm) containing 8 mL of 1X Hoagland solution (Hoagland and Arnon, 1950) supplemented with 0.625% (w/v) agar, and maintained at 4°C under dark conditions for 48 h (stratification). After stratification seeds were transferred to a growth chamber at 25°C, under a 16 h/8 h day/night photoperiod with a photosynthetically active radiation (PAR) of 60 μmol m^-2^ s^-1^.

Twenty eight days after germination, seedlings were randomly selected and transferred to plastic pots and grown hydroponically in a substrate mixture of vermiculite:perlite (2:1) for another 28 days, in a growth chamber under the described conditions. Four seedlings were placed in each pot and 3 sets of a total of 6 pots were treated with 1x Hoagland solution supplemented with 0 (control), 12.5 and 25 mg L^-1^ of metalaxyl (supplied as a fungicide solution commercially available as Ridomil^®^ by Syngenta Agro S.A., Portugal). Metalaxyl concentrations were selected based on preliminary data obtained from biometric parameters of plants exposed to: 0, 12.5, 25, 50, 75, 100 mg L^-1^. The concentrations chosen to conduct this study (12.5- and 25 mg L^-1^) allowed us to: (1) assess the differential physiological and biochemical responses of plants to the agrochemical, and (2), produce enough biomass for all assays. At the end of the treatment, plants were harvested and separated into roots and leaves and fully expanded intact leaves at the third leaf node from the apex were immediately used for biochemical determinations, or grounded to a fine powder in liquid nitrogen (N_2_) and stored at -80°C for further studies. The data represent the average of four independent replicates.

### Electrolyte Leakage

Leaves and roots were washed several times with deionized water and, after drying; tissue samples were immersed in 10 mL of deionized water and incubated at 25°C in a rotary shaker (100 rpm). Electrical conductivity of the bathing solution (T1) was recorded after 24 h. Samples were immediately autoclaved at 120°C for 20 min and a last conductivity reading (T2) was obtained when the solutions reached 25°C. EL was expressed following the formula: [I (%) = [1-(1-T1-T2)/(1-(C1-C2))] × 100], where C corresponds to the control situations and T to the treated samples.

### Superoxide Anion Radical ($O_{2}^{•–}$) Content and Histochemical Detection

Superoxide production was determined by a spectrophometric method based on the nitrobluetetrazolium (NBT) assay, where formazan is produced upon the reduction of NBT by superoxide (Gajewska and Skłodowska, 2007). A total of 0.1 g of *S. nigrum* leaves and roots (fresh material) was immersed in 0.01M of sodium phosphate buffer pH = 7.8, containing 0.05% (w/v) of NBT and 0.01M of sodium azide (NaN_3_). Samples were placed for 60 min in a rotary shaker (250 rpm), in the dark, at room temperature. After the reaction mixture was heated at 85°C for 15 min and cooled in an ice bath during 10 min, the A_580_ was measured. The reducing activity of NBT was expressed as the increase in the A_580_ per gram of fresh weight. Histochemical detection of $O_{2}^{•–}$ was performed in leaves of control and metalaxyl-treated plants according to (Romero-Puertas et al., 2004).

### Enzymes – Extraction and Assay Procedures

Frozen tissues (0.1 g) from *S. nigrum* roots and leaves were homogenized at 4°C with a MagNALyser (Roche, Vilvoorde, Belgium) in 1 mL of extraction buffer. Superoxide dismutase (SOD, EC1.15.1.1) and catalase (CAT, EC1.11.1.6) were extracted in a 50 mM potassium phosphate (pH 7.0) containing 0.4 mM EDTA, 0.2 mM PMSF, 2% (w/v) insoluble polyvinylpolypyrrolidone (PVPP) and 1 mM ascorbic acid. Other enzymes such as ascorbate peroxidase (APX, EC1.11.1.11), monodehydroascorbate reductase (MDHAR, EC1.6.5.4), dehydroascorbate reductase (DHAR, EC1.8.5.1), glutathione reductase (GR, EC1.8.1.7) and phenol peroxidase (GPOX, EC1.11.1.7) were extracted in 50 mM MES/KOH buffer (pH 6.0), containing 2 mM CaCl_2_, 40 mM KCl and 1 mM ascorbic acid. Ribulose 1,6-biphosphate carboxylase/oxygenase (RuB EC 4.1.1.39) was isolated from rye leaves using a modified method as described by (Wang and Portis, 1992). SOD activity was assayed in a 1.0 ml reaction mixture containing 50 mM potassium phosphate (pH 7.8) buffer, 13 mM methionine, 75 M nitro blue tetrazolium (NBT), 0.1 mM EDTA and 2 M riboflavin. Its activity was measured by the inhibition of the photochemical reduction of NBT at 560 nm, as described by (Dhindsa et al., 1981). CAT activity was determined spectrophotometrically at 240 nm by monitoring the rate of H_2_O_2_ decomposition at pH 7.0 (Aebi, 1984). APX, MDHAR, DHAR and GR activities were determined according to (Murshed et al., 2008). APX activity was measured in reaction mixtures consisting of 50 mM potassium phosphate buffer (pH 7.0), 0.25 mM AsA, and 5 mM H_2_O_2_. Activity was determined by measuring the decrease in reaction rate at A_290_. The fraction buffer of DHAR activity was 50 mM HEPES buffer (pH 7.0), contained 0.1 mM EDTA, 2.5 mM GSH, 0.2 mM DHA, and the activity was determined by measuring the increase in reaction rate at A_265_. MDHAR and GR activities were measured in HEPES buffer (100 mM, pH 7.6) and HEPES buffer (100 mM, pH 8), respectively. Their activities were determined by measuring the decrease in absorption at A_340_ and calculated from the 6.22 Mm^-1^_._cm^-1^ extinction coefficient. Peroxidase (POX) activity was determined by the oxidation of pyrogallol (𝜀_340_ = 2.47 mM^-1^ cm^-1^) (Kumar and Khan, 1982). The assay mixture of POX contained 0.05M phosphate buffer (pH 6.8), 0.01 M pyrogallol, 10 μl of 20 μL of enzyme extract and 0.01 M H_2_O_2_. Activity was determined by measuring the decrease in absorption at A_430_ and calculated from the 2.47 Mm^-1^cm^-1^. Soluble protein content for was obtained according to Lowry et al. (1951). RuBisCO was determined spectrophotometrically at 340 nm by monitoring the rate of NADH oxidation (Racker, 1962). The reaction mixture (1 mL) contained 1 M Tris-HCl (pH 7.8), 6 mM NADH, 0.1 M GSH, 0.5% (w/v) glyceraldehyde-3-phosphate, 25 mM 3-phosphoglycerate kinase, 0.05% (w/v) α-glycro-phosphate dehydrogenase-triose phosphate isomerase, 25 mM RuBP, 0.2 M ATP, 0.5 M MgCl_2_, 0.5 M KHCO and isolated RuBisCO extract. One unit of enzyme activity was defined as the amount of enzyme producing 1 μM of RuBisCO per min. All activity measurements were scaled down for semi-high throughput measurement using a micro-plate reader (Synergy Mx, Biotech Instruments Inc., Winooski, VT, United States). The photorespiration enzyme glycolate oxidase (GO, EC1.1.3.1) was determined according to (Feierabend and Beevers, 1972). GO was measured by the formation of a glyoxylate complex with phenylhydrazine (𝜀324 = 17 mM^-1^ cm^-1^).

### Non-enzymatic Antioxidants

#### Determination of Ascorbate and Glutathione Levels

Ascorbate (ASC) and glutathione (GSH) were determined by HPLC analysis (Potters et al., 2004). Frozen tissue was extracted in 1 ml of ice-cold 6% (w/v) meta-phosphoric acid and antioxidants were separated by reversed-phase HPLC (100 mm X 4.6 mm Polaris C18-A, 3 lm particle size; 40°C, isocratic flow rate: 1 mL min^-1^, elution buffer: 2 mM KCl, pH 2.5 with *O*-phosphoric acid). Antioxidants were quantified using a custom made electrochemical detector and the purity and identity of the peaks were confirmed using an in-line DAD (SPDM10AVP, Shimadzu). Total antioxidant concentration was determined after reduction with 40 mM dithiothreitol (DTT) and the redox status was calculated as the ratio of the reduced form and the total concentration of the antioxidant.

#### Determination of Total Antioxidant Capacity

A modified phosphomolybdenum assay (PM) was used to estimate the total antioxidant capacity (TAC) of plant extracts. A 0.1 mL extract and 1 mL of the *Reagent Solution* (0.6 M sulfuric acid, 28 mM sodium phosphate and 4 mM ammonium molybdate) were combined. The absorbance of the reaction mixture was measured at 695 nm, and TAC was expressed as ascorbic acid equivalents (AsA) per mg of fresh weight. Ascorbic acid was prepared in methanol 95 % (v/v) and used as reference standard.

#### Total Phenolic Content

Total phenolic content (TPC) was quantitatively estimated in both leaves and roots of plants (fresh material) using the Folin-CioCalteu reagent according to (Velioglu et al., 1998). Samples were measured in a spectrophotometer at 625 nm. Gallic acid was used as a reference standard for plotting calibration curve (0–20 μg/mL) and TPC was expressed as Gallic acid equivalents (GAE) per mg of fresh weight.

#### Total Flavonoids Content

Total flavonoid content (TFC) was quantitatively determined using the aluminum chloride colorimetric assay as described by (Kamal, 2011). Standard quercetin solutions (0–100 μg/mL) were used to plot a calibration curve and, based on the measured absorbance (A_415_), TFC was expressed as quercetin equivalents (QE) per mg of fresh weight.

#### Tocopherol Content

Tocopherols were determined by HPLC analysis according to (AbdElgawad et al., 2015). Tocopherols were extracted with hexane using the MagNALyser (Roche, Vilvoorde, Belgium; 1 min, 7000 rpm). The dried extract (CentriVap concentrator, Labconco, KS, United States) was resuspended in hexane, and tocopherols were separated and quantified by HPLC (Shimadzu, ‘s Hertogenbosch, The Netherlands) (normal phase conditions, Particil Pac 5 μm column material, length 250 mm, i.d. 4.6 mm). Dimethyl tocol (DMT) was used as internal standard (5 ppm). Data were analyzed with Shimadzu Class VP 6.14 software.

### Soluble Protein, SDS-PAGE and Relative Rubisco Content

Total soluble protein in the leaves was determined by the Bradford method (Lowry et al., 1951). SDS-PAGE of soluble proteins was performed as described by (Laemmli, 1970) and the gels were stained with Coomassie Brilliant Blue R250. Relative RuBisCO content was determined according to Soares et al. (2016) after extraction of rubisco small and large subunit bands in formamide. Rubisco content was expressed relative to total protein content, after extraction and spectrophometric determination of all proteins in the gel.

### Carbohydrate Extraction and Analysis

The soluble sugar content was analyzed by the anthrone method (Irigoyen et al., 1992). Briefly, 0.2 g (FW) of plant material (leaves and roots) was extracted in 80% ethanol at 80°C for 60 min. Total soluble sugars were analyzed by adding 3 mL of freshly prepared anthrone reagent (150 mg anthrone in 100 mL 72% H_2_SO_4_) to 0.1 mL of the alcoholic extract. This mixture was placed in a water bath at 100°C for 10 min and then cooled in an ice bath for 5 min. The absorbance of samples was measured at 625 nm. The sugar content was calculated from a standard curve prepared with known concentrations of D-glucose in a range of 0–100 μg mL^-1^. Following the extraction of soluble sugars, the solid fraction was used for the extraction of starch in 30% perchloric acid at 60°C for 60 min. In this procedure 0.3 mL of the acidic extract were added to 3 mL of anthrone. Test tubes were placed in a water bath at 90°C for 10 min and cooled down in an ice bath until the mixture reached room temperature. D-glucose was also used as standard (in a range of 0–100 μg mL^-1^) and the optical density of the starch solution read at 625 nm (Osaki et al., 1991).

### Free Amino Acid Measurements

Ethanolic root and shoot extracts of *S. nigrum* plants were used for assaying free amino acids (FAAs) levels using a Waters Acquity UPLC-tqd system (Milford, MA, United States) equipped with a BEH amide 2.1 × 50 column according to (Sinha et al., 2013) with the minor modification previously described by (AbdElgawad et al., 2015).

### Metalaxyl Levels

Metalaxyl levels were determined in both organs of 25 mg L^-1^-treated plants as previously described (Teixeira et al., 2011).

### Statistical Analysis

Results were expressed as mean ± SD (standard deviation) and analyzed by two-way ANOVA using IBM SPSS Statistica 23 software package (SPSS^®^ Inc; Chicago, IL, United States) for windows, with organs and concentrations used as fixed variables. Data were tested for normal distribution and homogeneity and normalized when necessary. A significance level of 0.05 was used for rejection of the null-hypothesis. In cases of significant interactions between factors, one-way ANOVA analysis was performed for each factor, and Tukey’s multiple range test was used to determine significant differences among means. All experiments were carried out in quadruplicate (*n* = 4).

## Results

### Total Biomass and Internal Concentrations of Metalaxyl

**FIGURE 1:**
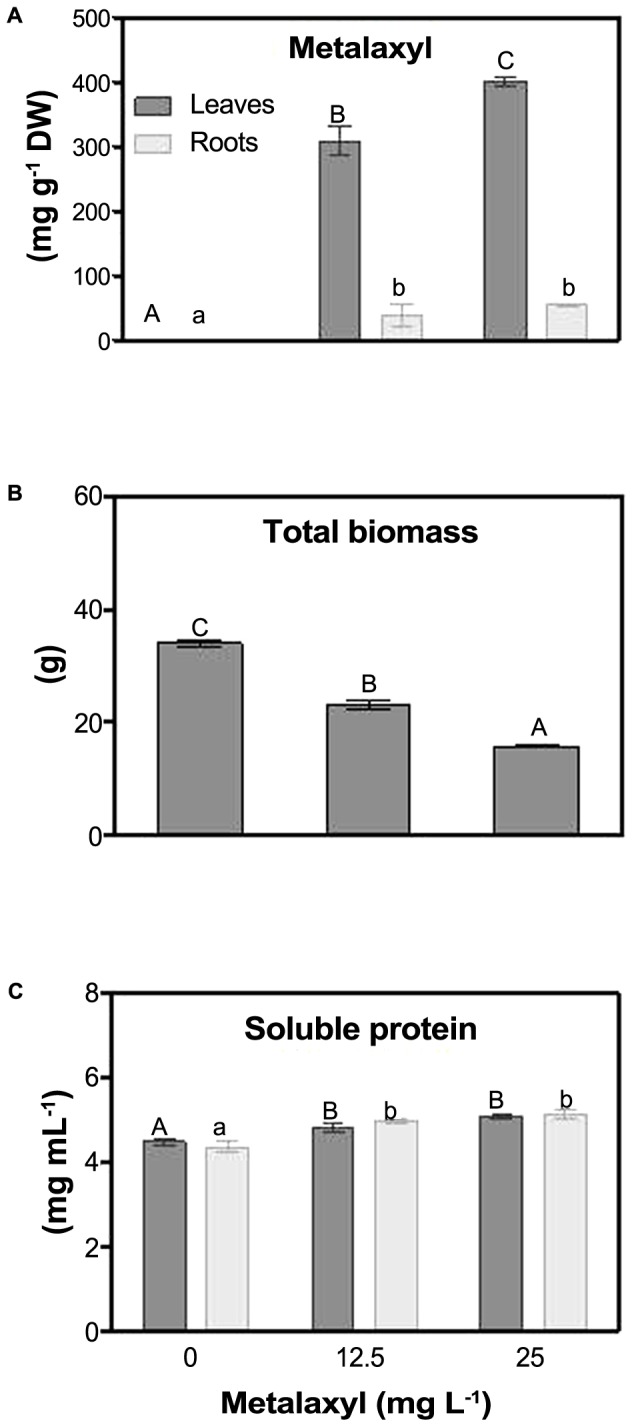
Metalaxyl levels (adapted from Teixeira et al., 2011) **(A)**, total biomass **(B)** and soluble protein content **(C)** in leaves and roots of *S. nigrum* plants supplemented with 0, 12.5 and 25 mg L^-1^ of the fungicide. Columns represent mean ± SEM (*n* = 4).

As shown in **Figure 1**, 25 mg L^-1^-treated plants accumulate metalaxyl, with leaves containing seven times more metalaxyl than roots (**Figure 1A**). Total biomass of 25 mg L^-1^- treated plants decreased 50%, when compared to control plants (**Figure 1B**). Total soluble protein content increased in both roots and shoots in plants treated with the lowest concentration of the fungicide (**Figure 1C**).

### Photosynthetic-Related Processes

**FIGURE 2:**
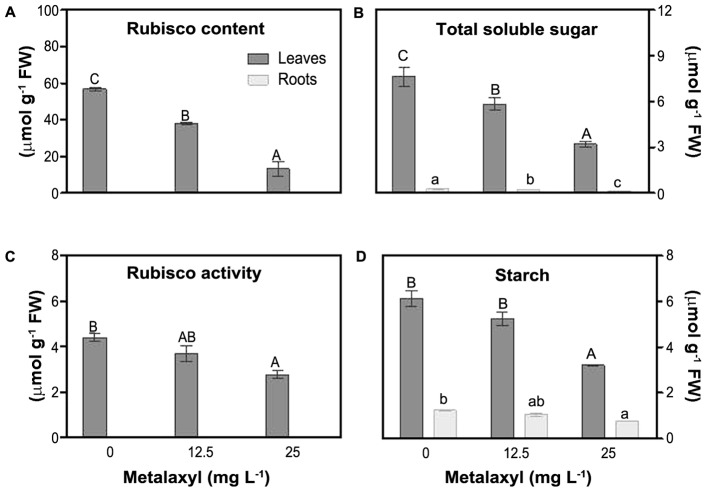
Rubisco relative content **(A)**, total soluble sugar (TSS) **(B)**, rubisco activity **(C)** and starch **(D)** measurements in leaves and roots of *S. nigrum* plants supplemented with metalaxyl (0, 12.5, and 25 mg L^-1^). Columns represent mean ± SEM (*n* = 4).

To estimate impact on plant photosynthetic capacity, we measured rubisco activity and rubisco content (relative to soluble protein content), as well as soluble sugar and starch content (**Figure 2**). Relative rubisco content (%) decreased by 28 and 46% in leaves of plants exposed to 12.5 and 25 mg L^-1^ of metalaxyl, respectively (**Figure 2A**) and rubisco activity decreased by 16 and 37% (**Figure 2C**). Leaf and root carbohydrate and starch levels decreased under stress. TSS and starch levels decreased as the concentration of metalaxyl increased for both leaves and roots (**Figures 2B,D**).

### Oxidative Stress Indicators

**FIGURE 3:**
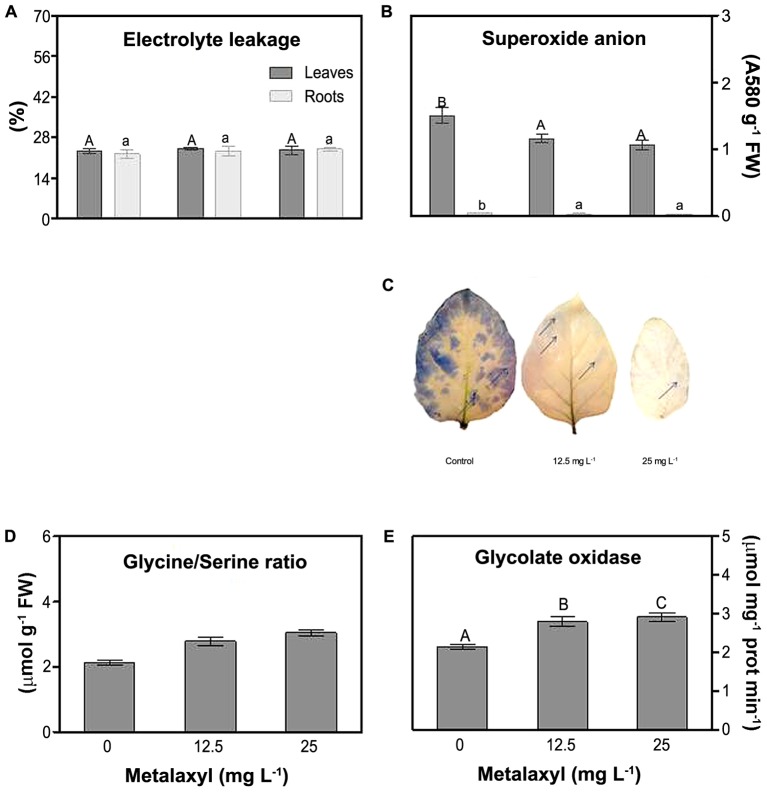
Electrolyte leakage **(A)**, superoxide anion content **(B)**, histochemical detection of superoxide anion **(C)** Gly/Ser **(D)** and glycolate oxidase **(E)** measurements *S. nigrum* plants supplemented with (0, 12.5, and 25 mg L^-1^). Columns represent mean ± SEM (*n* = 4).

Various parameters were measured to determine the oxidative stress status of the metalaxyl-treated plants. First, electrolyte leakage (EL) did not present significant fluctuations for both roots and shoots at all metalaxyl concentrations tested (**Figure 3A**). Superoxide was determined spectrophotometrically in leave and root extracts (**Figure 3B**), and through histochemical staining (**Figure 3C**) of plants supplemented with 0, 12.5, and 25 mg L^-1^ metalaxyl. Leaves contain considerably higher $O_{2}^{•–}$, compared to roots. This ROS significantly decreased by 29 and 39% in shoots and roots, respectively, in 25 mg L^-1^- treated plants (**Figure 3B**). Histochemical detection in leaves confirmed these results, with leaves of control plants showing higher $O_{2}^{•–}$-induced staining (blades and margins), compared to leaves of metalaxyl-treated plants (**Figure 3C**). As another indicator of oxidative stress-related changes, we estimated leaf photorespiration, which is an important source of hydrogen peroxide. Increased Gly/Ser ratios (11 and 18% increase) and higher GO activity (11 and 9% increase) were observed in leaves after exposure to 12.5- and 25 mg L^-1^ of metalaxyl respectively (**Figures 3D,E**).

### Antioxidant Metabolites and Enzymes

**FIGURE 4:**
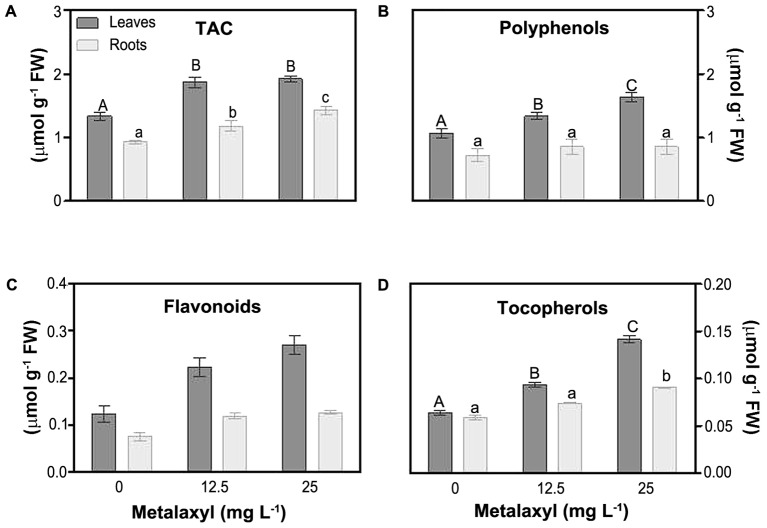
Total antioxidant activity (TAC) **(A)** and non-enzymatic antioxidant levels [phenols **(B)**, flavonoids **(C)** and tocopherols **(D)** in leaves and roots of *S. nigrum* plants supplemented with metalaxyl (0, 12.5, and 25 mg L^-1^). Columns represent mean ± SEM (*n* = 4).

Molecular and enzymatic antioxidants, protect plants largely against ROS impact. These are partially organized in the ASC/GSH cycle (see below). One estimate of the molecular capacity to scavenge cellular ROS is through determination of the total antioxidant capacity (TAC). This parameter significantly increased, in leaves (about 40%), and roots (by 27 and 53%), in response to metalaxyl (**Figure 4A**). As the TAC assay only measures overall changes in the small molecular antioxidant pool, predominant antioxidant, i.e., polyphenols, flavonoids, tocopherols, ascorbate and gluthatione, were separately quantified. Polyphenol levels were higher in leaves then in roots, and were significantly induced in leaves under the metalaxyl treatment (**Figure 4B**). Other plant phenols, such as flavonoids, also act as powerful antioxidants. Flavonoid levels were about 49 and 87% in leaves of 12.5- and 25 mg L^-1^- treated plants, respectively. Roots behaved in a similar way, with increases of 45 and 62% (**Figure 4C**). Tocopherol, protecting against lipid oxidation, levels were higher in leaves than in roots, and increased in the metalaxyl treatment (45 and 70% for leaves) (**Figure 4D**).

**FIGURE 5:**
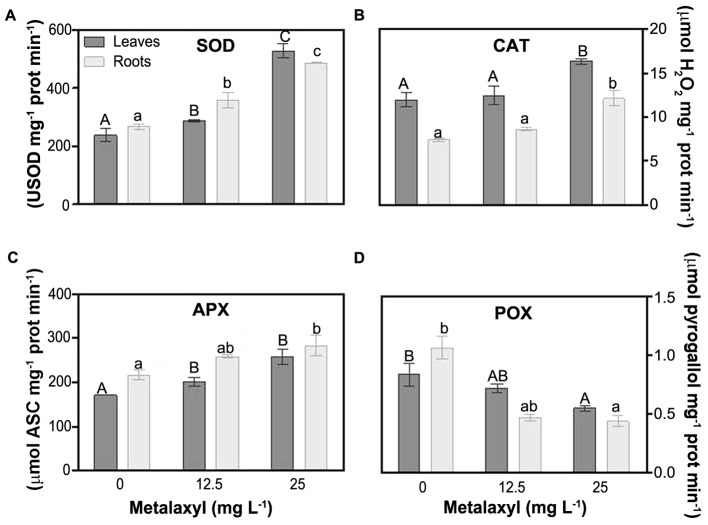
Response of ROS scavenging enzymes in leaves and roots of *S. nigrum* plants supplemented with metalaxyl (0, 12.5, and 25 mg L^-1^). Superoxide dismutase (SOD) **(A)**, catalase (CAT) **(B)**, ascorbate peroxidase (APX) **(C)** and peroxidase (POX) **(D)**. Columns represent mean ± SEM (*n* = 4).

Among the enzymatic antioxidants, SOD is essential in controlling $O_{2}^{•–}$ levels. Metalaxyl increased SOD activity in leaves (21 and 83%) and roots (34 and 35%) (**Figure 5A**). Several enzymes, are dedicated to H_2_O_2_ scavenging. CAT activity was always higher in roots than in leaves, independent of the experimental situation. Metalaxyl exposure, increased CAT in roots (73% in 25 mg L^-1^), and leaves (37%) (**Figure 5B**). Other enzymes important in controlling H_2_O_2_ levels are the peroxidases. General peroxidase activity (**Figure 5D**), was not induced by metalaxyl exposure, but rather decreased, in leaves (14 and 24%) and roots (56 and 7%). Another enzyme involved in defense against H_2_O_2_ damage, APX, is a key enzyme in the ASC-GSH cycle. It is supplied with electrons by oxidation of ascorbate, which is recycled in a series of redox reactions. Plants exposed to the highest concentration of the pesticide exhibited a significant increase in APX activity of 51 and 33% in leaves and roots, respectively (**Figure 5C**).

**FIGURE 6:**
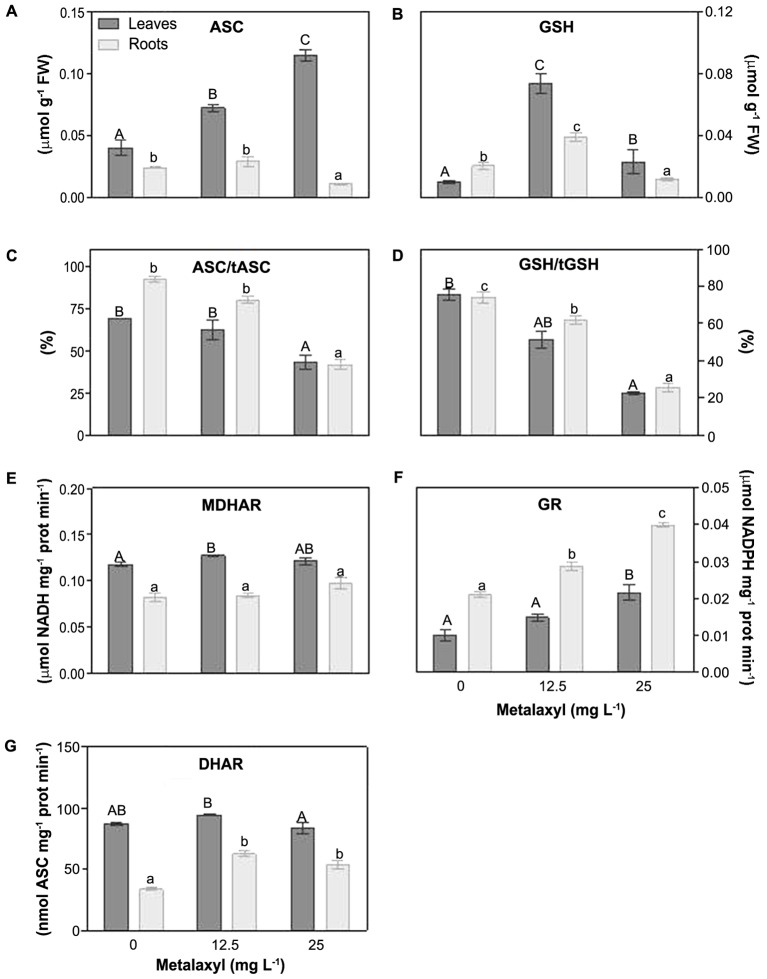
Response of the ASC/GSH cycle components in leaves and roots of *S. nigrum* plants, supplemented with metalaxyl (0, 12.5 and 25 mg L^-1^). Reduced ascorbate levels (ASC) **(A)**, reduced gluthatione levels (GSH) **(B)**, ascorbate redox status **(C)**, gluthatione redox status **(D)**, MDHAR activity **(E)**, GR activity **(F)** and DHAR activity **(G)**. Different uppercase letters represent significant differences between treatments in leaves, while lowercase letters represent significant differences between treatments in roots. Values represent mean ± SD (*n* = 4) (*P* ≤ 0.05).

Other parameters of the ASC-GSH cycle, include the ascorbate and glutathione levels and redox status, and the recycling enzymes, MDHAR, DHAR, GR were measured (**Figures 6A–E**). With respect to ASC, its levels were increased in leaves (59 and 81%), but reduced in roots by 62% in 25 mg L^-1^-treated plants (**Figure 6A**). The ascorbate redox status decreased in leaves and roots (**Figure 6C**). MDHAR remains relatively unchanged (**Figure 6E**), whereas DHAR increased slightly, predominantly at 25 mg L^-1^ (**Figure 6G**). Also the GSH concentration was increased predominantly at 25 mg L^-1^ (**Figure 6B**). The glutathione redox status, decreased in leaves and roots (**Figure 6D**), whereas the GR activity increased in leaves and roots (**Figure 6F**).

## Discussion

Previously it was shown that metalaxyl accumulated in leaves of *S. nigrum* plants, generating a mild oxidative stress, and increased glutathione peroxidase (GPX), glutathione-S transferase (GST), and the non-enzymatic antioxidant Pro (Teixeira et al., 2011). To gain a broader understanding of how metalaxyl exposure affects metabolic processes, we analyzed its effects on photosynthesis, photorespiration, sugar accumulation and antioxidant defenses.

### Metalaxyl Inhibited Growth by Reducing Photosynthesis and Inducing Photorespiration

Rubisco can be a rate-limiting enzyme for photosynthesis, especially when plants are exposed to stressful conditions (Wingler et al., 2000). Here, we observe that metalaxyl decreased rubisco activity and amount, probably leading to the inhibition of photosynthesis and abrupt decrease in plant growth. Consistently, the lower content of TSS and starch in metalaxyl-treated leaves reflects the decrease in photosynthesis. Under metalaxyl exposure, we also observed increases in the photorespiration parameters Gly/Ser ratio and glycolate oxidase (GO) activity. Photorespiratory rates increase as a result of the oxygenase activity of rubisco at low CO_2_ and high O_2_ conditions. The increased photorespiration, strongly indicates elevated cellular levels of H_2_O_2_, which is also consistent with previous results (Teixeira et al., 2011).

Our results suggest that metalaxyl treatment affected the water status of *S. nigrum* plants, since leaves of treated plants showed a tendency to wilt that became more severe in plants treated with the highest concentration of the fungicide (25 mg L^-1^). Consistently, in a previous work, we observed a significant increase in proline levels which is widely known to act as an osmoprotectant in water-stressed plants (Teixeira et al., 2011). This proteinogenic amino acid may also contribute to reducing the oxidative damage induced by metalaxyl.

### Induced Antioxidant Defense Protects Metalaxyl-Treated Plants from Oxidative Damage

To inspect for oxidative stress signs, we investigated membrane integrity (electrolyte leakage), and $O_{2}^{•–}$ levels. Notably, there was no apparent increase in membrane damage, indicating that the stress exposure was at least not very severe. This is consistent with our previous finding of reduced MDA levels in metalaxyl-treated plants (Teixeira et al., 2011). This considerable resistance is remarkably consistent with the bioremediation capacity of this species. Also consistent with the lack in membrane damage, are the lowered $O_{2}^{•–}$ levels, which in turn can be explained by the enhancement of the antioxidant defenses. In fact, SOD is described as a first line of defense against ROS and higher SOD activity often appears to enhance plant tolerance to oxidative damage induced by multiple biotic and abiotic stress factors (Gill and Tuteja, 2010). Our previous studies showed that metalaxyl stimulated the accumulation of hydrogen peroxide (H_2_O_2_) in shoots and roots of *S. nigrum*, at similar metalaxyl concentrations. Therefore, as a consequence, the ratio of $O_{2}^{•–}$/H_2_O_2_ decreases in metalaxyl-treated plants, lowering the rate production of OH^-^, probably also contributing to reduced oxidative damage.

Finally, reduced membrane damage observed in metalaxyl-treated plants could also be explained by enhanced tocopherols levels observed under stress conditions. These lipophilic membrane-embeded antioxidants, prevent chain propagation in lipid peroxidation and efficiently scavenge singlet oxygen radicals (^1^O_2_). Consistent with the increased tocopherols, metalaxyl exposure also induced significant increases in total antioxidant capacity (TAC), polyphenols and flavonoid contents, especially in leaves. This suggests that non-enzymatic antioxidants may contribute considerably in helping leaf tissues accumulating higher metalaxyl levels with few collateral effects on the oxidative metabolism.

Along with the non-enzymatic antioxidants, H_2_O_2_-scavenging enzymes are involved in the fine tuning H_2_O_2_ levels. Catalase (CAT) is a peroxisomal matrix enzyme protecting peroxisomal proteins and membranes from oxidative damage (Mhamdi et al., 2010). CAT activity significantly increased in *S. nigrum* plants submitted to 25 mg L^-1^ of metalaxyl. CAT activity may be regulated by H_2_O_2_ levels. Here, we found that enhanced H_2_O_2_ levels quantified in previous studies (Teixeira et al., 2011) induced antioxidant protection by increasing CAT catalytic activity. This behavior can be interpreted as a compensatory mechanism to confront the mild oxidative stress imposed by metalaxyl treatment in plants. Similar results were found in suspension cells of *S. nigrum* propagated in 20- and 40 mg L^-1^ of metalaxyl (de Sousa et al., 2013). In addition to CAT, also POX and APX activities contribute significantly to H_2_O_2_-level control. Peroxidases are heme-containing glycoproteins encoded by a large multigene family, involved in several physiological processes and are well known to play a key role in oxidative stress conditions (Kawano, 2003). Some peroxidases use aromatic electron donors as substrates to reduce H_2_O_2_ (Hiraga et al., 2001). POX activities were reduced after metalaxyl exposure in both roots and shoots of *S. nigrum*. This suggests that this enzyme does not play a pivotal role in defense mechanisms against mild oxidative stress induced by the fungicide.

### Metalaxyl-Induced Responses of the ASC-GSH Cycle in *S. nigrum* Plants

The ASC/GSH cycle is a combination of reactions in which APX reduces H_2_O_2_ at the expense of reduced ascorbate. Oxidized ascorbate is regenerated through MDHAR, DHAR and GR, using electrons from GSH and NADPH (Foyer and Noctor, 2011). Although ASC plays several physiological roles, its function in the ASC/GSH cycle puts it central in the maintenance of the cellular redox status. Under metalaxyl treatment, MDHAR and DHAR showed only moderate increases, somewhat more pronounced in roots. We also observed a decreasing ascorbate redox status in metalaxyl exposure, which is consistent with the mild oxidative stress in the plants, and a largely unaltered recycling capacity (Teixeira et al., 2011). As for GSH, the GSH pool increased at mild metalaxyl doses. However, the glutathione redox status decreased despite an increase in GR activity. The decreased GSH levels and redox status indicate relatively high glutathione consumption levels. In particular at the higher metalaxyl concentrations, this GSH consumption could also be related to elevated toxin conjugation through GST activity. This hypothesis is in agreement with the increased root GST activity reported previously in *S. nigrum* (Teixeira et al., 2011).

## Conclusion

The results presented here show that the fungicide metalaxyl limits *S. nigrum* growth due to impairment of photosynthesis, which results in disturbance of sugar accumulation and allocation. However, no severe oxidative damage was observed in metalaxyl-treated plants, which can be explained by the efficient maintenance of low ROS levels provided by antioxidant enzymes and antioxidant metabolites. This capacity to control oxidative stress is most probably contributing significantly to the capacity of *S. nigrum* to act as a phytoremediator.

## Author Contributions

AdS and HAE equally designed and contributed to the experimental procedures and writing of the manuscript. HA, FF, JT, AP, CS, SB-N, TB, SS, SAJ, and MA helped in the experimental design, contributed to experimental procedures and reviewed the manuscript.

## Conflict of Interest Statement

The authors declare that the research was conducted in the absence of any commercial or financial relationships that could be construed as a potential conflict of interest.
